# Characteristics and Outcomes of Patients Injured in Road Traffic Crashes and Transported by Emergency Medical Services

**DOI:** 10.3390/ijerph13020236

**Published:** 2016-02-19

**Authors:** Chun-Ying Huang, Cheng-Shyuan Rau, Jung-Fang Chuang, Pao-Jen Kuo, Shiun-Yuan Hsu, Yi-Chun Chen, Hsiao-Yun Hsieh, Ching-Hua Hsieh

**Affiliations:** 1 Department of Trauma Surgery, Kaohsiung Chang Gung Memorial Hospital and Chang Gung University College of Medicine, Kaohsiung 833, Taiwan; junyinhuang@yahoo.com.tw (C.-Y.H.); jjfce0624@gmail.com (J.-F.C.); ah.lucy@hotmail.com (S.-Y.H.); libe320@yahoo.com.tw (Y.-C.C.); sylvia19870714@hotmail.com (H.-Y.H.); 2 Department of Neurosurgery, Kaohsiung Chang Gung Memorial Hospital and Chang Gung University College of Medicine, Kaohsiung 833, Taiwan; ersh2127@cloud.cgmh.org.tw; 3 Department of Plastic and Reconstructive Surgery, Kaohsiung Chang Gung Memorial Hospital and Chang Gung University College of Medicine, Kaohsiung 833, Taiwan; bow110470@gmail.com

**Keywords:** emergency medical services (EMS), injury severity score (ISS), mortality

## Abstract

To investigate the injury characteristics and mortality of patients transported by emergency medical services (EMS) and hospitalized for trauma following a road traffic crash, data obtained from the Trauma Registry System were retrospectively reviewed for trauma admissions between 1 January 2009 and 31 December 2013 in a Level I trauma center. Of 16,548 registered patients, 3978 and 1440 patients injured in road traffic crashes were transported to the emergency department by EMS and non-EMS, respectively. Patients transported by EMS had lower Glasgow coma scale (GCS) scores and worse hemodynamic measures. Compared to patients transported by non-EMS, more patients transported by EMS required procedures (intubation, chest tube insertion, and blood transfusion) at the emergency department. They also sustained a higher injury severity, as measured by the injury severity score (ISS) and the new injury severity score (NISS). Lastly, in-hospital mortality was higher among the EMS than the non-EMS group (1.8% *vs.* 0.3%, respectively; *p* < 0.001). However, we found no statistically significant difference in the adjusted odds ratio (AOR) for mortality among patients transported by EMS after adjustment for ISS (AOR 4.9, 95% CI 0.33–2.26), indicating that the higher incidence of mortality was likely attributed to the patients’ higher injury severity. In addition, after propensity score matching, logistic regression of 58 well-matched pairs did not show a significant influence of transportation by EMS on mortality (OR: 0.578, 95% CI: 0.132–2.541 *p* = 0.468).

## 1. Background

Emergency medical services (EMS) are responsible for transporting victims of motor vehicle accidents to local emergency departments. Burt *et al.* reported that approximately 82% of injured patients were brought to emergency departments and trauma centers by means other than the ambulance [1]. However, another study reported that only close to 10% of injured patients arrived at trauma centers by private vehicle [2].

Although pre-hospital EMS play a major role in any trauma system, some studies have suggested that certain interventions performed by EMS providers might delay transport to definitive care, or might be associated with worsened outcomes, or both [3,4,5]. Existing data have suggested that private vehicle transport of injured patients may be associated with improved survival compared with transport by EMS [6]. However, their study did not have enough statistical power to detect a difference in mortality rates [6]. In a retrospective cohort study of state trauma registry data of patients admitted to all Pennsylvania trauma centers over five years, overall injury severity and mortality were higher among patients transported by EMS than among those transported by private vehicle [2]. Even after adjusting for injury severity, patients transported by EMS were twice as likely to die as patients transported by private vehicle [2]. The debate regarding the association of EMS transportation with higher mortality is yet to be settled, particularly as it is commonly believed that more highly trained EMS providers are able to care better for injured patients, are more readily able to identify acute illness, and can transport patients to the hospital faster than a private person.

In the United States, EMS may be fire department- or hospital-based, whereas in Taiwan, EMS are exclusively fire department-based. In Taiwan, ground transportation is typically the only means of transportation in both rural and urban areas, with few exceptions (accidents or disasters occurring on a mountain or on the sea) [7]. Although there are some private ambulance companies in Taiwan, they mostly assist with non-emergencies, transfers of stable patients between hospitals, or the transportation of the dead. In the past, EMS in Taiwan have focused on transporting patients to hospitals. In 1995, Taiwan’s Congress passed EMS laws to regulate EMS providers’ licensing and mandated that every local fire department had to set up emergency medical technician (EMT)-I, -II, and -P (paramedic) teams in its jurisdiction. The EMT-1 provides vital sign measurement, basic life support (BLS) skills, and automated external defibrillator (AED) operation. The EMT-2 level provides additional electrocardiography (ECG) monitoring, application of the laryngeal mask and pneumatic anti-shock garment, and some medications, e.g., intravenous saline or oral glucose water supplement [8]. Furthermore, the EMT-P can provide advanced life support (ALS) [8]. However, limited information about the involvement of EMS in transporting patients injured in motor vehicle accidents in Taiwan is available. To address this issue, our aim was to describe the characteristics and outcomes of patients injured in road traffic crashes and transported to the hospital by EMS. We compared the outcomes of patients transported by EMS (EMS group) with the outcomes of those transported by friends, relatives, bystanders, or who drove themselves (non-EMS group) in a Level I trauma center over a five-year period using data from a population-based trauma registry.

## 2. Methods

### Study Design

The study was conducted at the Kaohsiung Chang Gung Memorial Hospital, a 2400-bed facility and Level I regional trauma center that provides care to trauma patients primarily from South Taiwan. Approval for this study was obtained from the institutional review board (IRB) of Chang Gung Memorial Hospital with the approval number 103-4088B before its initiation. An informed consent was waived according to the regulation of IRB.

This retrospective study was designed to review all 16,548 hospitalized and registered patients added to the Trauma Registry System from 1 January 2009 to 31 December 2013, and select cases that met the following inclusion criteria: (1) involvement in a traffic crash; (2) transportation by EMS or non-EMS vehicles (including friends, relatives, bystanders, and patients driving themselves; not including police) and (3) admittance due to the traffic crashes. The exclusion criteria included: (1) patients who were transferred from other hospitals because their condition was generally stable after the examinations or procedures that had been performed in the previous hospital; and (2) incomplete registered data. During this time, 5729 trauma patients were transported to the hospital by EMS and 5436 patients by non-EMS. Of these patients, a total of 3978 (69.4%) were involved in a traffic crash and transported to the emergency department by EMS. For comparison, data on 1440 (26.5%) patients transported by non-EMS were also collected.

Detailed patient information was retrieved from the Trauma Registry System of our institution and included data regarding the transfer by EMS, age, sex, vital signs on arrival, the Glasgow coma scale (GCS) score assessed on arrival at the emergency department, details of procedures performed at the emergency department (intubation, chest tube insertion, and blood transfusion), abbreviated injury scale (AIS) scores for each body region, injury severity scores (ISS), new injury severity scores (NISS), trauma and injury severity scores (TRISS), length of stay (LOS) in the hospital, LOS in the intensive care unit (ICU LOS), in-hospital mortality, and complications associated with the injuries.

Adjusted odds ratios (AORs) and 95% confidence intervals (CI) for mortality stratified by ISS were calculated. A blood alcohol concentration (BAC) level of 50 mg/dL or higher at the time of arrival at the hospital was defined as positive based on the legal limit for drivers in Taiwan. In our study, the primary outcomes were injury severity as measured by different scoring system (GCS, AIS, ISS, NISS, and TRISS) and in-hospital mortality. The secondary outcomes were LOS and ICU LOS.

Data were compared using the SPSS v. 20 statistical software (IBM Corporation, Armonk, NY, USA). We used the Pearson’s chi-squared test, Fisher’s exact test, or independent Student’s *t*-test, as applicable. To minimize confounding effects due to nonrandomized assignment in the assessment of mortality, propensity scores were calculated using a logistic regression model and the following covariates: age, alcohol intoxication (Alcohol > 50 mg/dL), GCS, injury to the regions of head/neck, thorax, abdomen, or extremity based on AIS, and ISS. A 1:1 matched study group was created by the Greedy method with NCSS software (NCSS 10, NCSS Statistical software, Kaysville, UT, USA). After amending these confounding factors, binary logistic regression was used in the evaluation of interventional factor of obesity on mortality. All results are presented as means ± standard errors. A *p*-value of <0.05 was considered as statistically significant.

## 3. Results

### Patient Characteristics

The mean ages of the 3978 patients transported by EMS and 1440 by non-EMS following a road traffic crash were 44.1 ± 19.3 and 43.6 ± 20.1 years, respectively (Table 1). Fewer patients aged 0–9 years were noted among those transported by EMS than non-EMS (1.0% *vs.* 2.6%, respectively; *p* < 0.001). A statistically significant difference regarding sex was found between patients transported by EMS (2220 [55.8%] men and 1758 [44.2%] women) and patients transported by non-EMS (849 [59.0%] men and 591 [41.0%] women). Most injured patients were motorcyclists; among these, 81.5% (*n* = 3243) were transported by EMS and 74.2% (*n* = 1068) by non-EMS. There were significantly more motorcyclists among patients transported by EMS than among those transported by non-EMS (*p* < 0.001). In contrast, significantly more bicyclists were transported by non-EMS than by EMS. The majority of patients in both groups (EMS and non-EMS) arrived at the emergency department between 5 p.m. and 11 p.m.; however, more patients transported by EMS than non-EMS arrived during this time period. In contrast, more patients transported by non-EMS than EMS arrived between 7 a.m. and 5 p.m. A positive BAC was more frequent among patients transported by EMS than those transported by non-EMS (10.0% *vs.* 5.0%, respectively; *p* < 0.001). Of the 3978 patients transported by EMS, the transport times (18.3 ± 7.9 min, range 2–89 min) and procedures performed by the EMT were recorded for 3813 (95.9%) patients (Table 2).

We found a significant difference in GCS scores (14.1 ± 2.4 *vs.* 14.8 ± 1.2, respectively; *p* < 0.001) and the distribution of scores among the patients (GCS ≤ 8, 9–12, or ≥13) between the EMS and non-EMS groups (Table 3). Patients transported by EMS had lower GCS scores than those transported by non-EMS, and a higher proportion of patients with a GCS score of either ≤8 or between 9–12 were found among this group. Our analysis of AIS scores revealed that patients transported by EMS had sustained significantly higher rates of injuries to the head/neck, face, thorax, and abdomen, while patients transported by non-EMS sustained significantly higher rates injuries to the extremities. The comparison of trauma injury scores between the EMS and non-EMS groups indicated significant differences regarding the ISS (9.7 ± 7.7 *vs.* 6.4 ± 4.5, respectively; *p* < 0.001). When stratified by injury severity (ISS of <16, 16–24, or ≥25), more patients in the EMS than the non-EMS group had an ISS between 16 and 24 or an ISS of ≥25. In contrast, more patients transported by non-EMS than EMS had an ISS of <16. Likewise, we also found significant differences regarding the NISS (11.4 ± 9.2 *vs.* 7.1 ± 5.2, respectively; *p* < 0.001), TRISS (0.960 ± 0.112 *vs.* 0.979 ± 0.069, respectively; *p* < 0.001), and in-hospital mortality rates (1.8% *vs.* 0.3%, respectively; *p* < 0.001) between the two patient groups. However, after adjustment for ISS, we found no significantly different adjusted odds ratio (AOR) for patient mortality between the patients transported by EMS (AOR 4.9, 95% CI 0.33–2.26). In addition, after a 1:1 propensity score matching, 58 well-balanced pairs of patients were available for outcome comparison (Table 4) and logistic regression analysis did not show a significant influence of transportation by EMS on mortality (OR: 0.578, 95% CI: 0.132–2.541 *p* = 0.468), indicating that the differences in injury severity, but not the transportation method, between the two groups of patients may have been responsible for their different mortality rates.

Patients transported by EMS exhibited higher odds ratios (ORs) for presenting with worse hemodynamic measures than patients transported by non-EMS. These measures included a systolic blood pressure (SBP) of <90 mmHg, heart rate of >100 beats/min, respiratory rate of <10 or >29 times/min, and shock index of >0.9 (Table 4). In addition, patients transported by EMS had higher odds for requiring procedures at the emergency department, including intubation, chest tube insertion, and blood transfusion.

The findings regarding the injuries associated with the road traffic crashes are listed in the Supplementary Table S1. In summary, the patients transported by EMS had statistically significantly higher ORs for sustaining certain types of head/neck trauma (cranial fractures, epidural hematomas, subdural hematomas, subarachnoid hemorrhages, intracerebral hematomas, and cerebral contusions), maxillofacial trauma (maxillary and nasal fractures), thoracic trauma (hemothorax, lung contusions), abdominal trauma (intra-abdominal and hepatic injuries), and extremity trauma (humeral, pelvic, femoral, tibial, and fibular fractures). In contrast, they had significantly lower odds of sustaining clavicle, radial, or metacarpal fractures.

As shown in Table 5, a significantly longer LOS in the hospital was found among patients transported by EMS than among those transported by private vehicle (9.6 days *vs.* 6.1 days, respectively; *p* < 0.001). When stratified by injury severity, a significantly longer LOS in the hospital was found in the EMS than the non-EMS group for patients with an ISS of <16 (8.2 days *vs.* 5.9 days, respectively; *p* < 0.001) and between 16 and 24 (14.4 days *vs.* 10.0 days, respectively; *p* = 0.008). No statistically significant difference in the LOS in the hospital was found for patients with an ISS of ≥25 between the two groups. A significantly larger proportion of patients transported by EMS was admitted to the ICU (18.9% *vs.* 5.0%, respectively; *p* < 0.001) regardless of stratification into either group of injury severity (ISS of <16, 16–24, or ≥25). However, there was no significant difference between EMS and non-EMS patients regarding the LOS in the ICU.

Further analysis of the EMS patients revealed the mortality of 73 patients and the survival of 3905 patients (Table 6). The mean ages of the EMS patients who died and those who survived were 57.0 ± 19.7 and 43.9 ± 19.2 years, respectively (*p* < 0.001). No statistically significant difference regarding sex or transport times was found between the death and the survivors. A positive BAC was more frequent among the EMS patients who died than those who survived (21.9% *vs.* 9.9%, respectively; *p* = 0.001). The comparison of trauma injury scores between the dead and the survivors indicated significant differences regarding the ISS (32.9 ± 18.1 *vs.* 9.3 ± 6.6, respectively; *p* < 0.001). When stratified by injury severity, more of the EMS patients who died had an ISS between 16 and 24 or an ISS of ≥25 than ones who survived. By contrast, more of the surviving EMS patients had an ISS of <16. Regarding the injuries commonly associated with mortality, the EMS patients who died had statistically significantly higher ORs for epidural hematomas, subdural hematomas, subarachnoid hemorrhages, cerebral contusions, hemothorax, pneumothorax, hemopneumothorax, hepatic injuries, and retroperitoneal injury. However, they had no significantly higher odds of sustaining splenic injury, renal injury, pelvic fracture, and femoral fracture.

## 4. Discussion

This study analyzed patient demographics and the characteristics of injuries observed in the trauma population transported to a Level I trauma center by EMS *vs.* non-EMS following road traffic crashes. Patients transported by EMS not only had a higher injury severity but also presented with worse physiological conditions (lower GCS scores, deteriorated vital signs, and higher shock index; all of which were statistically significant). In addition, procedures including intubation, chest tube insertion, and blood transfusion were more frequently performed in the EMS than the non-EMS group. Patients transported by EMS presented with a bodily injury pattern that differed from that of patients transported by non-EMS. Higher rates of injuries to the head/neck, face, thorax, and abdomen were found in patients transported by EMS, while patients transported by non-EMS sustained significantly higher rates of injuries to their extremities. The EMS group also had significantly higher odds of sustaining a variety of injuries but lower odds of sustaining clavicle, radial, and metacarpal fractures. Notably, patients transported by EMS tended to have injuries making it hard for them to drive themselves to the hospital (e.g., higher BACs, an unstable hemodynamic status, a variety of traumatic brain injuries, hemothorax, lung contusions, or lower limb fractures). In contrast, injuries to the extremities (e.g., fractures of the clavicle, radius, or metacarpus) may impair patients’ ability to drive themselves to a lesser extent. Our results are generally in agreement with those reported in the literature that overall injury severity is higher in patients transported by EMS [2,6], indicating those severely-injured patients may prefer a transportation by EMS. Moreover, the death EMS patients were older, tend to have a positive BAC, and as expected, were injured severely and had higher ORs of most of the common associated lethal illness.

With a much larger decline in prehospital than in-hospital trauma deaths after the implementation of the statewide EMS system, the EMS have been suggested to contributed to a significant decline in the number of trauma deaths [9]. The establishment of the state trauma system is associated with a 9% reduction in the risk of death caused by motor vehicle crash [10]. In contrast, some authors had reported a 1.6-fold higher in-hospital mortality of the patients transported by EMS, even after adjustment by injury severity (*p* = 0.002) [6]. Subgroup analysis showed that among patients with an ISS of >15, those in the EMS group had a mortality rate twice that of those in the non-EMS group (28.8% *vs.* 14.1%, respectively) [6]. Other reports similarly showed that mortality was higher among patients transported by EMS than among those transported by private vehicle [1,5]. However, in a prospective cohort-matched observation study, mortality, complications, and length of hospital stay were similar between the EMS- and non-EMS-transported groups [11]. In our study, we found no significantly different AOR for mortality among patients transported by EMS after the adjustment for ISS. The further comparison of 58 well-matched pairs of fatal patients after propensity score matching also did not show a significant influence of transportation by EMS on mortality. This indicates that the difference in injury severity may be responsible for the higher mortality rates and the involvement of EMS in this current condition did not decrease the mortality of these injured patients. However, given the limitations of a retrospective study of the registry data, our results should be interpreted with caution, particularly in consideration of the different trauma system and environmental factors. First, regarding the role of advanced life support (ALS) provided by EMS, it has also been reported that 76.3% of patients being transported by EMS received ALS and 23.7% basic life support (BLS) care in the United States [2]. Patients transported by EMS and receiving ALS and BLS pre-hospital care were more likely to die than those who were transported by private vehicle (ALS: OR 2.0, 95% CI 1.6–2.5; BLS: OR 1.6, 95% CI 1.2–2.0) after adjusting for injury severity [2]. According to an assessment of 12,502 EMS calls in urban Taipei, Taiwan, only 7.41% of the patients received ALS cases (ECG monitoring accounted for 3.13%, IV injections for 0.73%, and cardiopulmonary resuscitation (CPR) for 3.55%) [12]. In general, the ALS demand was estimated at around 9%–16% of EMS calls in Taipei [13,14]. The lower incidence of ALS demand may be attributed partly to fewer EMT-Ps in Taiwan (only 310 EMT-Ps across the country in 2008) [8], and partly to relatively less severe injuries in a crowded city, with motorcycle accidents predominant [15,16]. This fact may be reflected by a lower percentage of EMS personnel performing ALS on patients than prior reports [13,14]; only nine (0.2%) EMS patients received CPR, and no EMS patient received intubation. In addition, the in-hospital mortality rates for patients transported by EMS (1.8%) and non-EMS (0.3%) in our study were markedly lower than those reported in the literature. Second, it was reported previously that increased EMS pre-hospital time adversely affects patient mortality after motor vehicle accidents [2,17,18,19]. Critically injured patients (with an ISS of ≥13) transported by non-EMS arrived at the trauma center faster than those transported by EMS (15 min *vs.* 28 min, respectively; *p* < 0.05) [11]. Swift transport is particularly beneficial for neurotrauma and hemodynamically unstable penetratingly injured patients [20]. According to an evaluation carried out in Taipei, the response time of EMS was around 4 min on average [21] and the average transport time was about 12 min according to government data from January 2009 to June 2009 [7]. In this study, ALS *vs.* BLS does not make a difference especially when the field interventions are limited, but short transport time, prevention of secondary injuries, and basic life support may improve the survival of patients with severe injuries, who might have a poor outcome if transported through other means. However, the lack of recorded data for patients transported by non-EMS and response times (the time period from notification to arrival at the scene of a road traffic crash) impedes the further analysis of pre-hospital care. Considering that the majority of motorcycles are forbidden on highways in Asian cities and that most traffic crashes occur in relatively crowded streets in these cities, the question of whether the discrepancy between our findings and those of prior studies can be attributed to the involvement of EMS *per se* or to the relatively low velocity in most motorcycle injuries in the Asian region requires further investigation. The number of fatalities in the two groups of patients was too small to perform a powerful statistical analysis from which conclusions could have been drawn. Finally, the severely-injured patients who did not survive until arrival at the hospital and those patients who received ALS but were transferred from other hospitals were not included in the sample, thus probably making the number of ALS cases transported by EMS lower than expected. Clearly, the association between pre-hospital care by EMS and outcomes for injured patients is not well understood yet.

Our analysis has several limitations. First, our data were collected prospectively as part of the required trauma registry process, but our questionnaires and analyses were performed retrospectively and are thus subject to the limitations of all retrospective studies. Second, the process of selecting hospitals by EMS providers in trauma systems is affected by several factors other than field triage protocols [22]. In this study, the overall higher injury severity in patients transported by EMS suggested that severely injured patients prefer transportation by EMS; however, the lack of available data regarding the circumstances of the injury and the factors influencing the decision-making regarding a transfer to the emergency department by EMS *vs.* non-EMS may result in a bias, particularly as the study population was limited to a single urban trauma center. Third, injured patients who did not survive until arrival at the hospital or who were discharged from the emergency department were not included in the sample. It is conceivable that civilians typically do not obey pre-hospital trauma triage guidelines and may preferentially transfer injured patients to the closest hospital, and not necessarily a trauma center. In addition, death outside of our study sample is more prominent among non-EMS transports, resulting in a survival bias. Lastly, the impact of pre-existing comorbidities in the studied populations on the hospitalization course and mortality remains unclear.

### Strengths and Limitations of This Study

- Patients transported by EMS had lower GCS scores, worse hemodynamic measures, and higher injury severity.
- More required procedures (intubation, chest tube insertion, and blood transfusion) at the emergency department were encountered in patients transported by EMS.
- Higher incidence of mortality in the patients transported by EMS was likely attributed to the patients’ higher injury severity.
- In this study, the lack of available data regarding the circumstances of the injury and the factors influencing the decision-making regarding a transfer to the emergency department by EMS *vs.* non-EMS may have led to selection bias.

## 5. Conclusions

Our analysis of the data on trauma admissions at a Level I trauma center spanning five years indicates that patients transported by EMS presented with a bodily injury pattern that differed from that of patients transported by non-EMS. Patients transported by EMS were shown to have a higher injury severity, worse outcomes, and higher mortality. However, no significant influence of transportation by EMS or non-EMS on mortality was found after adjustment for ISS or after propensity score matching, indicating that the higher mortality rates associated with EMS may be attributed to a difference in injury severity.

## Figures and Tables

**Table 1:** Demographics of patients transported by Emergency Medical Services (EMS) and non-EMS.

Variables	EMS, *n* (%)	Non-EMS, *n* (%)	Odds Ratio (95% CI)	*p*
	*N* = 3978	*N* = 1440		
Age	44.1 ± 19.3	43.6 ± 20.1		0.058
Age category (years)				
0–9	38 (1.0)	38 (2.6)	0.4 (0.23–0.56)	<0.001
10–19	440 (11.1)	166 (11.5)	1.0 (0.79–1.15)	0.630
20–29	725 (18.2)	241 (16.7)	1.1 (0.85–1.30)	0.206
30–39	480 (12.1)	177 (12.3)	1.0 (0.82–1.18)	0.822
40–49	562 (14.1)	189 (13.1)	1.1 (0.91–1.30)	0.345
50–59	747 (18.8)	274 (19.0)	1.0 (0.84–1.15)	0.836
60–69	588 (14.8)	201 (14.0)	1.1 (0.90–1.27)	0.448
70–79	305 (7.7)	122 (8.5)	0.9 (0.72–1.12)	0.331
80–89	86 (2.2)	32 (2.2)	1.0 (0.65–1.47)	0.893
≥90	7 (0.2)	0 (0.0)		0.111
Gender			0.9 (0.78–0.99)	0.039
Male	2220 (55.8)	849 (59.0)		
Female	1758 (44.2)	591 (41.0)		
Mechanism				
Driver of mobile	78 (2.0)	21 (1.5)	1.4 (0.83–2.20)	0.223
Passenger of mobile	42 (1.1)	13 (0.9)	1.2 (0.63–2.19)	0.620
Driver of motorcycle	3243 (81.5)	1068 (74.2)	1.5 (1.33–1.77)	<0.001
Passenger of motorcycle	217 (5.5)	91 (6.3)	0.9 (0.66–1.10)	0.225
Bicycle	235 (5.9)	202 (14.0)	0.4 (0.32–0.47)	<0.001
Pedestrian	159 (4.0)	45 (3.1)	1.3 (0.92–1.81)	0.136
Unspecific	4 (0.1)	0 (0.0)		0.229
Time				
7:00–17:00	607 (15.3)	256 (17.8)	0.8 (0.71–0.98)	0.025
17:00–23:00	2207 (55.5)	748 (51.9)	1.2 (1.02–1.30)	0.021
23:00–7:00	1164 (29.3)	436 (30.2)	1.0 (0.84–1.09)	0.499
BAC > 50 mg/dL	398 (10.0)	72 (5.0)	2.1 (1.64–2.74)	<0.001

**Table 2:** Transport time and procedures performed by emergency medical technicians (EMTs).

Variables	EMS, *n* (%) *N* = 3813
Transport time	
Mean (mins)	18.3 ± 7.9
Range (mins)	2–89
Procedures performed by EMT	
Intubation	0 (0)
Oxygenation	220 (5.8)
Cardiopulmonary resuscitation	9 (0.2)
Airway	35 (0.9)
Neck collar	1065 (27.9)
Backboard	1093 (28.7)
Spinal immobilizer	15 (0.4)
Splint	1104 (29.0)
Bandage	2581 (67.7)

**Table 3:** Injury characteristics, physiological status on arrival, and procedures performed at the emergency department of patients transported by Emergency Medical Services (EMS) and non-EMS.

Variables	EMS, *n* (%)	Non-EMS, *n* (%)	Odds Ratio	*p*
	*N* = 3978	*N* = 1440	(95% CI)	
Injury characteristics				
GCS	14.1 ± 2.4	14.8 ± 1.2		<0.001
≤8	229 (5.8)	16 (1.1)	5.4 (3.26–9.06)	<0.001
9–12	203 (5.1)	10 (0.7)	7.7 (4.06–14.55)	<0.001
≥13	3546 (89.1)	1414 (98.2)	0.2 (0.10–0.23)	<0.001
AIS				
Head/Neck	1407 (35.4)	230 (16.0)	2.9 (0.47–3.36)	<0.001
Face	912 (22.9)	228 (15.8)	1.6 (1.35–1.86)	<0.001
Thorax	647 (16.3)	187 (13.0)	1.3 (1.09–1.56)	0.003
Abdomen	268 (6.7)	73 (5.1)	1.4 (1.04–1.77)	0.026
Extremity	2965 (74.5)	1137 (79.0)	0.8 (0.67–0.90)	0.001
ISS	9.7 ± 7.7	6.4 ± 4.5		<0.001
<16	3266 (82.1)	1370 (95.1)	0.2 (0.18–0.30)	<0.001
16–24	499 (12.5)	56 (3.9)	3.5 (2.67–4.71)	<0.001
≥25	213 (5.4)	14 (1.0)	5.8 (3.34–9.93)	<0.001
NISS	11.4 ± 9.2	7.1 ± 5.2		<0.001
TRISS	0.960 ± 0.112	0.979 ± 0.069		<0.001
Mortality	73 (1.8)	5 (0.3)	5.4 (2.16–13.30)	<0.001
Physiology at ER, *n* (%)				
SBP < 90 mmHg	120 (3.0)	18 (0.6)	4.9 (3.01–8.14)	<0.001
Heart rate > 100 beats/min	719 (17.9)	293 (10.0)	1.9 (1.68–2.25)	<0.001
Respiratory rate <10 or >29	37 (0.9)	3 (0.1)	9.0 (2.78–29.25)	<0.001
Shock index > 0.9	254 (6.3)	68 (2.3)	2.8 (2.15–3.70)	<0.001
Procedures at ER, *n* (%)				
Intubation	170 (4.2)	4 (0.1)	32.1 (11.90–86.65)	<0.001
Chest tube insertion	65 (1.6)	21 (0.7)	2.3 (1.40–3.77)	0.001
Blood transfusion	158 (3.9)	15 (0.5)	7.9 (4.64–13.45)	<0.001

**Table 4:** Significant covariates of the patients transported by Emergency Medical Services (EMS) and non-EMS before and after propensity score matching (1:1 matching).

Variables	Before Matching				After Matching			
	Death *n* = 78	Survival *n* = 5340	Odds Ratio (95%)	*p*	Death *n* = 58	Survival *n* = 58	Odds Ratio (95%)	*p*
Age	56.6 ± 19.8	43.8 ± 19.5	-	<0.001	56.1 ± 19.7	54.8 ± 17.5	-	0.713
BAC > 50	16 (20.5)	478 (9.0)	2.6 (1.5–4.6)	<0.001	10 (17.2)	10 (17.2)	1.0 (0.4–2.6)	1.000
GCS	7.1 ± 4.4	14.4 ± 1.9	-	<0.001	7.9 ± 4.4	8.3 ± 4.6	-	0.611
AIS, *n* (%)								
Head/Neck	67 (85.9)	1570 (29.4)	14.6 (7.7–27.8)	<0.001	50 (86.2)	50 (86.2)	1.0 (0.4–2.9)	1.000
Thorax	33 (42.3)	801 (15.0)	4.2 (2.6–6.6)	<0.001	22 (37.9)	22 (37.9)	1.0 (0.5–2.1)	1.000
Abdomen	12 (15.4)	329 (6.2)	2.8 (1.5–5.2)	0.001	10 (17.2)	10 (17.2)	1.0 (0.4–2.6)	1.000
Extremity	36 (46.2)	4066 (76.1)	0.3 (0.2–0.4)	<0.001	29 (50.0)	29 (50.0)	1.0 (0.5–2.1)	1.000
ISS	32.3 ± 17.7	8.5 ± 6.2	-	<0.001	24.7 ± 8.0	24.6 ± 8.3	-	0.927

**Table 5:** Length of stay in the hospital and the intensive care unit (ICU) of patients transported by Emergency Medical Services (EMS) and non-EMS.

Variables	ISS	EMS *N* = 3978	Non-EMS *N* = 1440	Odds Ratio (95% CI)	*p*
Hospital LOS					
days		9.6 ± 9.7	6.1 ± 6.2		<0.001
	<16	8.2 ± 7.6	5.9 ± 6.0		<0.001
	16–24	14.4 ± 13.0	10.0 ± 6.8		0.008
	≥25	19.9 ± 17.5	14.6 ± 11.3		0.155
ICU LOS					
*n* (%)		750 (18.9)	72 (5.0)	4.4 (3.44–5.67)	<0.001
	<16	234 (7.2)	42 (3.1)	2.4 (1.75–3.41)	<0.001
	16–24	334 (66.9)	21 (37.5)	3.4 (1.90–5.98)	<0.001
	≥25	182 (85.4)	9 (64.3)	3.3 (1.03–10.38)	0.036
days		7.1 ± 9.0	5.8 ± 7.6		0.441

**Table 6:** Injury characteristics of death and survival patients that had been transported by Emergency Medical Services (EMS).

Variables	Death *n* = 73	Survival *n* = 3905	Odds Ratio (95% CI)	*p*
Age	57.0 ± 19.7	43.9 ± 19.2	–	<0.001
Gender				
Male	47 (64.4)	2173 (55.6)	1.4 (0.89–2.34)	0.136
Female	26 (35.6)	1732 (44.4)	0.7 (0.43–1.13)	0.136
Transport time				
Mean (mins)	21.8 ± 6.6	22.5 ± 9.6	–	0.520
Range (mins)	12–36	4–192	–	–
BAC > 50 mg/dL	16 (21.9)	386 (9.9)	2.6 (1.46–4.50)	0.001
ISS	32.9 ± 18.1	9.3 ± 6.6	–	<0.001
<16	5 (6.8)	3261 (83.5)	0.02 (0.01–0.04)	<0.001
16–24	15 (20.5)	484 (12.4)	1.8 (1.03–3.25)	0.037
≥25	53 (72.6)	160 (4.1)	62.0 (36.21–106.24)	<0.001
Diagnosis				
Epidural hematoma (EDH)	18 (24.7)	196 (5.0)	6.2 (3.57–10.75)	<0.001
Subdural hematoma (SDH)	39 (53.4)	399 (10.2)	10.1 (6.29–16.15)	<0.001
Subarachnoid hemorrhage (SAH)	34 (46.6)	485 (12.4)	6.1 (3.84–9.83)	<0.001
Intracerebral hematoma (ICH)	11 (15.1)	86 (2.2)	7.9 (4.01–15.49)	<0.001
Cerebral contusion	17 (23.3)	217 (5.6)	5.2 (2.95–9.03)	<0.001
Hemothorax	6 (8.2)	67 (1.7)	5.1 (2.15–12.24)	<0.001
Pneumothorax	4 (5.5)	76 (1.9)	2.9 (1.04–8.21)	0.033
Hemopneumothorax	5 (6.8)	47 (1.2)	6.0 (2.33–15.65)	<0.001
Hepatic injury	8 (11.0)	95 (2.4)	4.9 (2.30–10.58)	<0.001
Splenic injury	2 (2.7)	45 (1.2)	2.4 (0.58–10.15)	0.214
Retroperitoneal injury	1 (1.4)	6 (0.2)	9.0 (1.07–75.93)	0.014
Pelvic fracture	5 (6.8)	142 (3.6)	1.9 (0.77–4.91)	0.149
Femoral fracture	9 (12.3)	482 (12.3)	1.0 (0.49–2.02)	0.997

## Supplementary Materials

**Table S1:** Injuries of the patients transported by Emergency Medical Services (EMS) and non-EMS.

Variables	EMS, *n* (%) *N* = 3978		Non-EMS, *n* (%) *N* = 1440		Odds Ratio (95% CI)		*p*
Head/Neck trauma							
Neurologic deficit	28	(0.7)	6	(0.4)	1.7	(0.70–4.10)	0.237
Cranial fracture	323	(8.1)	47	(3.3)	2.6	(1.88–3.49)	<0.001
Epidural hematoma (EDH)	214	(5.4)	16	(1.1)	5.1	(3.05–8.48)	<0.001
Subdural hematoma (SDH)	438	(11.0)	35	(2.4)	5.0	(3.50–7.05)	<0.001
Subarachnoid hemorrhage (SAH)	519	(13.0)	50	(3.5)	4.2	(3.10–5.61)	<0.001
Intracerebral hematoma (ICH)	97	(2.4)	10	(0.7)	3.6	(1.86–6.87)	<0.001
Cerebral contusion	234	(5.9)	34	(2.4)	2.6	(1.79–3.72)	<0.001
Cervical vertebral fracture	24	(0.6)	6	(0.4)	1.5	(0.59–3.56)	0.413
Maxillofacial trauma							
Orbital fracture	88	(2.2)	23	(1.6)	1.4	(0.88–2.21)	0.158
Maxillary fracture	322	(8.1)	81	(5.6)	1.5	(1.15–1.90)	0.002
Mandibular fracture	100	(2.5)	25	(1.7)	1.5	(0.94–2.27)	0.092
Nasal fracture	60	(1.5)	7	(0.5)	3.1	(1.43–6.87)	0.003
Thoracic trauma							
Rib fracture	466	(11.7)	145	(10.1)	1.2	(0.97–1.44)	0.091
Sternal fracture	4	(0.1)	1	(0.1)	1.4	(0.16–12.97)	0.739
Hemothorax	73	(1.8)	15	(1.0)	1.8	(1.02–3.11)	0.041
Pneumothorax	80	(2.0)	22	(1.5)	1.3	(0.82–2.13)	0.248
Hemopneumothorax	52	(1.3)	15	(1.0)	1.3	(0.71–2.24)	0.435
Lung contusion	57	(1.4)	5	(0.3)	4.2	(1.67–10.43)	0.001
Thoracic vertebral fracture	26	(0.7)	6	(0.4)	1.6	(0.65–3.83)	0.315
Abdominal trauma							
Intra-abdominal injury	71	(1.8)	11	(0.8)	2.4	(1.25–4.47)	0.007
Hepatic injury	103	(2.6)	17	(1.2)	2.2	(1.33–3.73)	0.002
Splenic injury	47	(1.2)	19	(1.3)	0.9	(0.52–1.53)	0.683
Retroperitoneal injury	7	(0.2)	3	(0.2)	0.8	(0.22–3.27)	0.806
Renal injury	14	(0.4)	6	(0.4)	0.8	(0.32–2.20)	0.729
Urinary bladder injury	7	(0.2)	2	(0.1)	1.3	(0.26–6.11)	0.767
Lumbar vertebral fracture	42	(1.1)	13	(0.9)	1.2	(0.63–2.19)	0.620
Sacral vertebral fracture	24	(0.6)	4	(0.3)	2.2	(0.76–6.29)	0.140
Extremity trauma							
Scapular fracture	96	(2.4)	27	(1.9)	1.3	(0.84–1.99)	0.240
Clavicle fracture	495	(12.4)	229	(15.9)	0.8	(0.63–0.89)	0.001
Humeral fracture	213	(5.4)	15	(1.0)	5.4	(3.17–9.11)	<0.001
Radial fracture	400	(10.1)	203	(14.1)	0.7	(0.57–0.82)	<0.001
Ulnar fracture	205	(5.2)	87	(6.0)	0.8	(0.65–1.09)	0.201
Metacarpal fracture	118	(3.0)	74	(5.1)	0.6	(0.42–0.76)	<0.001
Pelvic fracture	147	(3.7)	12	(0.8)	4.6	(2.53–8.25)	<0.001
Femoral fracture	491	(12.3)	107	(7.4)	1.8	(1.41–2.19)	<0.001
Patella fracture	111	(2.8)	32	(2.2)	1.3	(0.85–1.88)	0.249
Tibial fracture	504	(12.7)	78	(5.4)	2.5	(1.98–3.24)	<0.001
Fibular fracture	295	(7.4)	36	(2.5)	3.1	(2.20–3.44)	<0.001
Calcaneal fracture	250	(6.3)	72	(5.0)	1.3	(0.98–1.68)	0.072
Metatarsal fracture	115	(2.9)	30	(2.1)	1.4	(0.93–2.10)	0.104

## Abbreviations

ALS: Advanced life support
AORs: Adjusted odd ratios
AIS: Abbreviated injury scale
BLS: Basic life support
BAC: Blood alcohol concentration
CI: Confidence intervals
EMS: Emergency medical services
EMT: Emergency medical technician
GCS: Glasgow coma scale
ICU: Intensive care unit
ISS: Injury severity score
LOS: Length of stay
NISS: New injury severity scores
SBP: Systolic blood pressure
TRISS: Trauma and injury severity scores

## References

[1] Burt C.W., McCaig L.F., Valverde R.H. (2006). Analysis of ambulance transports and diversions among US emergency departments. Ann. Emerg. Med..

[2] Johnson N.J., Carr B.G., Salhi R., Holena D.N., Wolff C., Band R.A. (2013). Characteristics and outcomes of injured patients presenting by private vehicle in a state trauma system. Am. J. Emerg. Med..

[3] Seamon M.J., Fisher C.A., Gaughan J., Lloyd M., Bradley K.M., Santora T.A., Pathak A.S., Goldberg A.J. (2007). Prehospital procedures before emergency department thoracotomy: “Scoop and run” saves lives. J. Trauma.

[4] Baez A.A., Lane P.L., Sorondo B., Giraldez E.M. (2006). Predictive effect of out-of-hospital time in outcomes of severely injured young adult and elderly patients. Prehospital Disaster Med..

[5] Osterwalder J.J. (2003). Mortality of blunt polytrauma: A comparison between emergency physicians and emergency medical technicians—Prospective cohort study at a level I hospital in eastern Switzerland. J. Trauma.

[6] Demetriades D., Chan L., Cornwell E., Belzberg H., Berne T.V., Asensio J., Chan D., Eckstein M., Alo K. (1996). Paramedic *vs.* private transportation of trauma patients. Effect on outcome. Arch. Surg..

[7] Hsiao K.Y., Lin L.C., Chou M.H., Chen C.C., Lee H.C., Foo N.P., Shiao C.J., Chen I.C., Hsiao C.T., Chen K.H. (2012). Outcomes of trauma patients: Direct transport *versus* transfer after stabilisation at another hospital. Injury.

[8] Chiang W.C., Ko P.C., Wang H.C., Yang C.W., Shih F.Y., Hsiung K.H., Ma M.H. (2009). EMS in Taiwan: Past, present, and future. Resuscitation.

[9] Ornato J.P., Craren E.J., Nelson N.M., Kimball K.F. (1985). Impact of improved emergency medical services and emergency trauma care on the reduction in mortality from trauma. J. Trauma.

[10] Nathens A.B., Jurkovich G.J., Rivara F.P., Maier R.V. (2000). Effectiveness of state trauma systems in reducing injury-related mortality: A national evaluation. J. Trauma.

[11] Cornwell E.E., Belzberg H., Hennigan K., Maxson C., Montoya G., Rosenbluth A., Velmahos G.C., Berne T.C., Demetriades D. (2000). Emergency medical services (EMS) *vs.* non-EMS transport of critically injured patients: A prospective evaluation. Arch. Surg..

[12] Hu S.C., Wang L.M. (1993). Study of patients arriving by ambulance in Taipei City. J. Formos. Med. Assoc..

[13] Lu T.C., Chen Y.T., Ko P.C., Lin C.H., Shih F.Y., Yen Z.S., Ma M.H., Chen S.C., Chen W.J., Lin F.Y. (2006). The demand for prehospital advanced life support and the appropriateness of dispatch in Taipei. Resuscitation.

[14] Hu S.C., Tsai J., Lu Y.L., Lan C.F. (1996). EMS characteristics in an Asian Metropolis. Am. J. Emerg. Med..

[15] Liang C.C., Liu H.T., Rau C.S., Hsu S.Y., Hsieh H.Y., Hsieh C.H. (2015). Motorcycle-related hospitalization of adolescents in a Level I trauma center in southern Taiwan: A cross-sectional study. BMC Pediatr..

[16] Liu H.T., Liang C.C., Rau C.S., Hsu S.Y., Hsieh C.H. (2015). Alcohol-related hospitalizations of adult motorcycle riders. WJES.

[17] Feero S., Hedges J.R., Simmons E., Irwin L. (1995). Does out-of-hospital EMS time affect trauma survival?. Am. J. Emerg. Med..

[18] Gonzalez R.P., Cummings G., Mulekar M., Rodning C.B. (2006). Increased mortality in rural vehicular trauma: Identifying contributing factors through data linkage. J. Trauma.

[19] Gonzalez R.P., Cummings G.R., Phelan H.A., Mulekar M.S., Rodning C.B. (2009). Does increased emergency medical services prehospital time affect patient mortality in rural motor vehicle crashes? A statewide analysis. Am. J. Surg..

[20] Harmsen A.M., Giannakopoulos G.F., Moerbeek P.R., Jansma E.P., Bonjer H.J., Bloemers F.W. (2015). The influence of prehospital time on trauma patients outcome: A systematic review. Injury.

[21] Ko P.C., Ma M.H., Yen Z.S., Shih C.L., Chen W.J., Lin F.Y. (2004). Impact of community-wide deployment of biphasic waveform automated external defibrillators on out-of-hospital cardiac arrest in Taipei. Resuscitation.

[22] Newgard C.D., Mann N.C., Hsia R.Y., Bulger E.M., Ma O.J., Staudenmayer K., Haukoos J.S., Sahni R., Kuppermann N. (2013). Patient choice in the selection of hospitals by 9-1-1 emergency medical services providers in trauma systems. Am. Emerg. Med..

